# Exploring the association between dietary vitamin A and coronary artery disease risk in men and women: findings from a US population study

**DOI:** 10.3389/fnut.2024.1418159

**Published:** 2024-10-15

**Authors:** Zhijian Wu, Weichang Yang, Haiyang Fang, Yi Chen, Yanqing Wu, Ren Gong

**Affiliations:** ^1^Department of Cardiology, The Second Affiliated Hospital of Nanchang University, Nanchang, China; ^2^Department of Respiratory Medicine, The Second Affiliated Hospital of Nanchang University, Nanchang, China; ^3^Cardiovascular and Cerebrovascular Disease Hospital Affiliated with Nanchang University, Nanchang, China

**Keywords:** coronary artery disease, dietary vitamin A, sex-difference, cross-sectional study, interaction

## Abstract

**Introduction:**

Coronary artery disease (CAD) is an important public health problem with negative impacts on individual health and socioeconomics. Studies on the relationship of dietary vitamin A (DVA) to CAD are limited and conflicting. The purpose of this study was to investigate the relationship between DVA and the prevalence of CAD in U.S. adults, with particular interest in sex differences.

**Methods:**

Data from 26,449 NHANES participants were used for analysis. The association of DVA with the prevalence of CAD was investigated utilizing multivariate logistic regression models and fitted smoothed curves, and interaction tests were performed to explore potential modifiers.

**Results:**

This study included 12,748 males and 13,701 females aged 50.34 ± 17.54 years. Overall, adjusted DVA was linearly negatively correlated with CAD (per natural ln (DVA) increment: OR 0.91, 95% CI 0.83–0.99). Multivariate regression analysis showed that among female participants, each natural increment of ln DVA was associated with a 22% reduction in CAD prevalence (OR 0.78, 95% CI 0.68–0.89). However, there was no significant correlation in male participants (*p* for interaction <0.001).

**Conclusion:**

DVA was negatively associated with the prevalence of CAD, and further analysis revealed an interaction between DVA and sex in terms of CAD prevalence.

## 1 Introduction

Individuals with coronary artery disease (CAD) are recognized as being at high or extremely high risk of developing adverse cardiovascular events in the future (1). The Global Burden of Disease International Collaborative Study published reports that CAD is the leading cause of death globally (2, 3). The number of deaths due to CAD is estimated to be 8.92 million globally, with an age-standardized mortality rate of 142/100,000, of which 173/100,000 are in the male population and 115/100,000 in the female population, with significant sex differences (2, 3). Besides revascularization, the treatment and prevention of CAD focuses on modifying cardiovascular risk factors, including lipid regulation to stabilize plaque, antiplatelet, control of blood pressure and blood glucose, and lifestyle improvements such as cessation of smoking and restriction of drinking, increase in physical activity, and improvement of dietary habits (4). Although guidelines exist, there is a relative dearth of therapeutic recommendations for the management of CAD patients to reduce cardiovascular risk factors. Dietary recommendations for patients with CAD commonly call only for a low-salt, low-fat diet and usually lack recommendations for specific nutrient intake. The World Health Organization encourages the intake of a variety of vegetables and fruits for vitamin supplementation to prevent cardiovascular disease but cannot recommend specific vitamins and intakes owing to a lack of clinical evidence (5).

Vitamin A is an essential nutrient that can be derived from both animal and plant foods and has many roles in gene regulation, embryonic development, skin health, and normal vision. There is evidence that vitamin A is a protective factor against cardiovascular disease (CVD) and all-cause mortality (6). Additionally, studies have shown that plasma retinol levels in patients with coronary artery disease are significantly lower than in healthy subjects (7, 8). However, interestingly, an early study showed that the beta-carotene and retinol-treated group had a 17% higher overall mortality rate and a 26% higher mortality rate from cardiovascular causes compared to the placebo group (9). Another study showed that participants taking vitamin A had significantly higher lipid levels than those in the placebo group, and it is well-known that dyslipidemia plays an important role in CAD (10).

Given the limited and conflicting research on the relationship between vitamin A and CAD, the effect of vitamin A on CAD is vague. With the aim of filling this knowledge gap, this study analyzes data from the 2007–2018 National Health and Nutrition Examination Survey (NHANES) to evaluate the effect of dietary vitamin A (DDA) on the prevalence of CAD and to explore potential factors that might modify this effect.

## 2 Methods

### 2.1 Study design and population

The NHANES is a cross-sectional investigation conducted biennially with a complex multi-stage sampling design. This survey aims to provide health and nutritional information for a representative sample of the US public. Ethical approval for the NHANES research program was obtained from the Ethics Review Board of the National Center for Health Statistics (NCHS). All individuals provided written informed consent prior to participation. For more information, visit www.cdc.gov/nchs/nhanes/index.htm.

**Figure 1:**
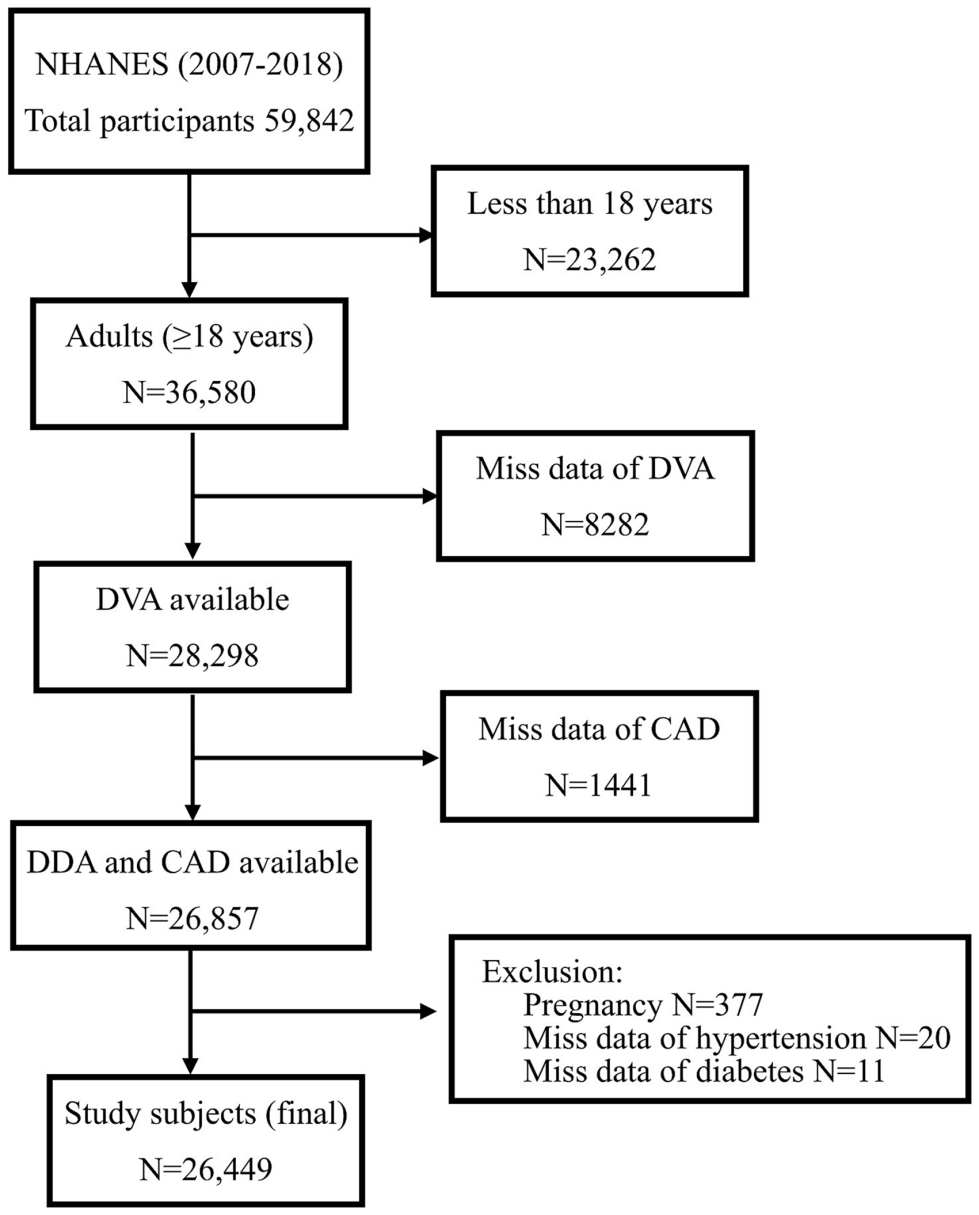
Flow chart of participants. DVA, dietary vitamin A; CAD, coronary artery disease.

The present study explored sex-specific differences in the relationship between DVA and CAD among US adults using data from 6 survey cycles of NHANES between 2007 and 2018. Overall, 59,842 individuals were recruited in the above cycle. Exclusion criteria were as follows: those younger than 18 years (*N* = 23,262), those who were in pregnancy (*N* = 377), those with missing DVA data (*N* = 8,282), and those with missing data on the history of CAD (*N* = 1,441), DM (*N* = 11), and HBP (*N* = 20). Lastly, 26,449 individuals were included in the analyses (Figure 1).

### 2.2 Dietary vitamin A

The nutritional assessment component occupies a very critical place in NHANES, a 24-h dietary recall interview conducted with participants of all ages by dietary interviewers who are professionally trained and fluent in Spanish and English. The first dietary recall interview was conducted instantly at the Mobile Examination Center (MEC), where subjects were interviewed about all food and beverage consumption within the past 24 h. The second interview was collected over the phone 3 to 10 days later. Standardized measurement tools were used to assist participants in accurately reporting the quantity and size of the food they consumed, followed by specialized software to calculate the total energy and nutrient intake of the foods. To provide a uniform measure of DVA, this study adopted the concept of retinol equivalents (RE), which is the amount of retinol corresponding to all vitamin A-active substances, such as retinol and β-carotene, as the intake of DVA. The total estimated value of DVA (μg/d) in this study is the average of the two recall periods.

### 2.3 Coronary artery disease

Similar to the previous NHANES study, the health status questionnaire in NHANES was administered by trained health professionals and each question was standardized. Subjects were administered a questionnaire by a professional and questioned about the following: “Has a doctor or other health care professional ever told you that you have CAD?,” “Has a doctor or other health care professional ever told you that you have angina pectoris?” and “Has a doctor or other health care provider ever told you about a heart attack? If their answer is “Yes” for at least one of the questions, they are assumed to have CAD (11, 12).

### 2.4 Potential covariates

Covariates that vary the DVK estimate of CAD over 10% or that are traditional risk factors for CAD were included in the final statistical analyses as potential confounders (13). After covariate screening, the following variables were used in the complete multivariate logistic regression analysis: Demographic data, including age, sex, race, educational attainment and poverty income ratio (PIR); dietary data for energy intake (kcal/d); examination data including body mass index (BMI, kg/m^2^), waist circumference (cm), and blood pressure (BP); laboratory data including alanine aminotransferase (ALT, U/L), serum creatinine (SCR, μmol/L), blood urea nitrogen (BUN, mg/dl), estimated glomerular filtration rate (eGFR, mL/min/1.73 m^2^), uric acid (UA, μmol/L), fasting blood glucose (FBG, mmol/L), glycosylated hemoglobin (HBA1c, %), total cholesterol (TC, mmol/L), and triglyceride (mmol/L); and questionnaire data including history of smoking, alcohol consumption, as well as with or without hypertension and diabetes. Additionally, the equation used to calculate eGFR is based on the modification of diet in the renal disease study group [MDRD, eGFR = 186 × (SCR × 0.011312) ^ (− 1.154) × age ^ (− 0.203) × (female × 0.762)] (14). Hypertension was defined as a self-reported diagnosis of hypertension by a healthcare practitioner, or systolic BP ≥140 mmHg and/or diastolic BP ≥90 mmHg (15). Diabetes was defined as a self-reported diagnosis of diabetes by a healthcare practitioner, or FBG ≥7 mmol/L, or HbA1c >6.5 (16).

### 2.5 Statistical analysis

Since DVA is skewed, this study log-transforms it with a constant e as the base (ln DVA) and then uses it for statistical analysis. Furthermore, it was grouped according to the DVA tertiles. To compare the baseline characteristics between groups, continuous data were analyzed by one-way ANOVA and categorical variables by chi-squared test or Fisher’s exact test. Continuous data were expressed as mean ± standard deviation or median (interquartile range), and categorical data were expressed as count (%). To assess the association of DVA with the prevalence of CAD, three multivariate regression models were constructed. In Model 1, no adjustment was made for any variables; in Model 2, demographic data including age, sex (only for all participants), race, education, and PIR were adjusted; in Model 3, BMI, waist circumference, smoking and drinking history, hypertension, diabetes, TC, triglycerides, UA, SCR, eGFR, BUN, ALT, and energy were adjusted additionally to the factors adjusted in Model 2. Using generalized additive modeling and smoothed curve fitting (penalized spline method) to visualize the shape of the association of DVA and CAD. Additionally, the potential effects of the following variables on the relationship between DVA and CAD were evaluated: age (<50 vs. 50–65 vs. >65 years), race (Mexican American vs. other Hispanic vs. non-Hispanic White vs. non-Hispanic Black vs. other race), educational attainment (<9th grade vs. 9–11th grade vs. high school vs. college vs. graduate and above), BMI (<25 vs. 25–30 vs. >30 kg/m^2^), smoking status (never vs. quit vs. current), alcohol consumption (never vs. 1–5 drinks/month vs. 5–10 drinks/month vs. >10 drinks/month vs. unknown), and eGFR (<60 vs. ≥60 mL/min/1.73 m^2^), hypertension (yes vs. no), diabetes (yes vs. no).

Missing values for variables were handled using the scientific method: continuous variables were interpolated using multiple interpolations, while categorical variables were handled by adding dummy variables [20.12% (*N* = 5,323) were missing for the history of drinking]. Generally, missing data were low (<10%) for all variables except drinking history. Comparisons of the supplemented data with the original data are shown in Supplementary Table S1. Statistical analyses were performed using the R package (version 4.2.1) and EmpowerStats (http://www.empowerstats.com, X&Y Solutions, Inc., Boston, MA). A two-tailed *p* < 0.05 was set for statistical significance.

## 3 Results

### 3.1 Study subjects’ baseline characteristics

Table 1 shows the baseline characteristics of the participants after grouping according to sex and DVA tertiles. Overall, there were 26,449 participants in this study, including 12,748 males and 13,701 females, with an age (mean ± standard deviation) of 50.34 ± 17.54 years. The prevalence of CAD was 7.53% (*N* = 1,991), generally consistent with the US Disease Survey (17), and the median DVA of participants was 506.5 μg/d (interquartile range 314.5–777.5 μg/d). Specifically, the prevalence of CAD and the median DVA for males were 9.84% and 536 μg/d (interquartile range 330.5–828.5 μg/d), respectively, whereas for females they were 5.37% and 480 μg/d (interquartile range 301.0–731.5 μg/d), respectively.

**Table 1:** Baseline characteristics of study participants.

Characteristics^a^^,^^b^	Males (*N* = 12,748)			*p*-value	Females (*N* = 13,701)			*p*-value
	Diatery vitamin A (μg/d) tertiles				Diatery vitamin A (μg/d) tertiles			
	Tertiles 1	Tertiles 2	Tertiles 3		Tertiles 1	Tertiles 2	Tertiles 3	
DVA range	0.0–396.0	396.5–710.5	711.0–23582.0		0.0–359.0	359.5–630.0	630.5–10475.5	
*N*	4,248	4,250	4,250		4,561	4,568	4,572	
Age, years	48.56 ± 17.25	51.06 ± 17.78	51.54 ± 17.88	<0.001	48.87 ± 17.10	50.32 ± 17.39	51.98 ± 17.62	<0.001
Race, %				<0.001				<0.001
Mexican American	715 (16.83%)	617 (14.52%)	472 (11.11%)		723 (15.85%)	727 (15.92%)	537 (11.75%)	
Other Hispanic	454 (10.69%)	398 (9.36%)	342 (8.05%)		558 (12.23%)	510 (11.16%)	430 (9.41%)	
Non-Hispanic White	1,457 (34.30%)	1,895 (44.59%)	2,241 (52.73%)		1,571 (34.44%)	1,956 (42.82%)	2,243 (49.06%)	
Non-Hispanic Black	1,135 (26.72%)	861 (20.26%)	723 (17.01%)		1,260 (27.63%)	914 (20.01%)	849 (18.57%)	
Other race	487 (11.46%)	479 (11.27%)	472 (11.11%)		449 (9.84%)	461 (10.09%)	513 (11.22%)	
Education level, %				<0.001				<0.001
<9th grade	576 (13.56%)	375 (8.82%)	265 (6.24%)		504 (11.05%)	429 (9.39%)	301 (6.58%)	
9–11th grade	739 (17.40%)	576 (13.55%)	467 (10.99%)		740 (16.22%)	570 (12.48%)	468 (10.24%)	
High school	1,122 (26.41%)	1,013 (23.84%)	923 (21.72%)		1,122 (24.60%)	1,024 (22.42%)	885 (19.36%)	
College	1,088 (25.61%)	1,194 (28.09%)	1,246 (29.32%)		1,431 (31.37%)	1,481 (32.42%)	1,498 (32.76%)	
Graduate or above	723 (17.02%)	1,092 (25.69%)	1,349 (31.74%)		764 (16.75%)	1,064 (23.29%)	1,420 (31.06%)	
PIR	2.29 ± 1.55	2.69 ± 1.63	2.86 ± 1.65	<0.001	2.16 ± 1.54	2.51 ± 1.62	2.72 ± 1.65	<0.001
Smoking status, %				<0.001				<0.001
Never smoking	1,833 (43.15%)	1,964 (46.21%)	2,117 (49.81%)		2,761 (60.53%)	2,932 (64.19%)	3,113 (68.09%)	
Quit smoking	1,098 (25.85%)	1,267 (29.81%)	1,301 (30.61%)		691 (15.15%)	859 (18.80%)	877 (19.18%)	
Current smoking	1,317 (31.00%)	1,019 (23.98%)	832 (19.58%)		1,109 (24.31%)	777 (17.01%)	582 (12.73%)	
Drinking status, %				0.30				0.011
Never drinking	565 (13.30%)	534 (12.56%)	578 (13.60%)		1,470 (32.23%)	1,388 (30.39%)	1,422 (31.10%)	
1–5 drinks/month	1,851 (43.57%)	1,878 (44.19%)	1,868 (43.95%)		1,596 (34.99%)	1,704 (37.30%)	1,631 (35.67%)	
5–10 drinks/month	355 (8.36%)	314 (7.39%)	353 (8.31%)		207 (4.54%)	210 (4.60%)	212 (4.64%)	
10+ drinks/month	660 (15.54%)	715 (16.82%)	642 (15.11%)		283 (6.20%)	320 (7.01%)	367 (8.03%)	
Unknown	817 (19.23%)	809 (19.04%)	809 (19.04%)		1,005 (22.03%)	946 (20.71%)	940 (20.56%)	
BMI, kg/m^2^	29.02 ± 6.30	29.20 ± 6.08	28.55 ± 6.08	<0.001	30.36 ± 7.74	29.95 ± 7.82	29.16 ± 7.53	<0.001
Waist circumference, cm	101.58 ± 16.23	102.69 ± 15.78	101.16 ± 15.76	<0.001	98.88 ± 16.87	98.34 ± 16.78	96.77 ± 16.61	<0.001
Hypertension, %				0.210				0.257
No	2,409 (56.71%)	2,292 (53.93%)	2,409 (56.68%)		2,542 (55.73%)	2,624 (57.44%)	2,587 (56.58%)	
Yes	1,839 (43.29%)	1,958 (46.07%)	1,841 (43.32%)		2,019 (44.27%)	1,944 (42.56%)	1,985 (43.42%)	
Diabetes, %				0.206				0.005
No	3,312 (77.97%)	3,333 (78.42%)	3,379 (79.51%)		3,628 (79.54%)	3,690 (80.78%)	3,760 (82.24%)	
Yes	936 (22.03%)	917 (21.58%)	871 (20.49%)		933 (20.46%)	878 (19.22%)	812 (17.76%)	
CAD				0.007				0.001
No	3,864 (90.96%)	3,783 (89.01%)	3,846 (90.49%)		4,270 (93.62%)	4,342 (95.05%)	4,353 (95.21%)	
Yes	384 (9.04%)	467 (10.99%)	404 (9.51%)		291 (6.38%)	226 (4.95%)	219 (4.79%)	
HBA1c, %	5.86 ± 1.19	5.81 ± 1.06	5.79 ± 1.07	0.019	5.79 ± 1.13	5.75 ± 1.02	5.73 ± 0.97	0.017
FBG, mmol/L	5.91 ± 2.40	5.81 ± 2.12	5.79 ± 2.17	0.046	5.65 ± 2.15	5.65 ± 2.18	5.53 ± 1.91	0.012
ALT, U/L	24.00 (18.00–33.00)	24.00 (19.00–33.00)	24.00 (18.00–32.00)	0.326	17.00 (14.00–23.00)	18.00 (15.00–23.00)	18.00 (15.00–24.00)	0.071
SCR, μmol/	91.89 ± 55.30	90.99 ± 37.24	90.31 ± 38.05	0.267	70.79 ± 37.14	69.37 ± 31.82	70.02 ± 36.36	0.159
BUN, mg/dL	4.94 ± 2.09	5.28 ± 2.18	5.43 ± 2.16	<0.001	4.56 ± 2.25	4.73 ± 2.06	4.91 ± 2.12	<0.001
eGFR, mL/min/1.73 m^2^	91.35 ± 25.54	89.88 ± 26.42	89.51 ± 23.35	0.003	94.98 ± 29.21	94.98 ± 28.32	92.84 ± 27.08	<0.001
UA, μmol/L	369.09 ± 80.29	363.60 ± 78.67	352.93 ± 77.68	<0.001	296.20 ± 80.65	290.78 ± 76.88	289.00 ± 76.87	<0.001
TC, mmol/L	4.93 ± 1.11	4.90 ± 1.11	4.85 ± 1.08	0.002	5.05 ± 1.09	5.06 ± 1.05	5.11 ± 1.05	0.021
Triglyceride, mmol/L	1.49 (0.97–2.34)	1.50 (1.00–2.35)	1.47 (0.96–2.28)	0.166	1.29 (0.87–1.90)	1.29 (0.88–1.94)	1.28 (0.86–1.92)	0.646
Energy, kcal/d	1907.29 ± 724.16	2279.66 ± 732.29	2703.21 ± 982.94	<0.001	1456.29 ± 529.74	1772.91 ± 562.70	2009.13 ± 663.56	<0.001

DVA, dietary vitamin A; PIR, poverty income ratio; BMI, body mass index; CAD, coronary artery disease; HbA1c, hemoglobin A1c; FPG, fasting plasma glucose; ALT, alanine aminotransferase; SCR, serum creatinine; BUN, blood urea nitrogen; eGFR, estimated glomerular filtration rate; UA, uric acid; TC, total cholesterol.

a Missing treatment: categorical variables (adding dummy variables), continuous variables (multiple interpolations).

b Data are presented as mean ± standard deviation, median (interquartile range), or numbers (%) as appropriate.

In male participants, compared to the low DVA population, the moderate to high DVA population were usually older, non-Hispanic White, and non-smokers had higher levels of educational attainment, PIR, BUN, and energy intake, had lower levels of BMI, waist circumference, HbA1c, FBG, eGFR, UA, and TC, and did not have CAD. Among female participants, compared with the low DVA population, those with moderate to high DVA tended to be older, non-Hispanic white, never-smokers, and drinkers; to have higher levels of education, PIR, BUN, TC, and energy intake; to have lower values of BMI, waist circumference, HbA1c, FBG, eGFR, and UA; and to be free of CAD and diabetes. However, there were no significant differences in the history of hypertension, ALT, SCR, and triglyceride levels among the three groups of male and female subjects. Moreover, no significant differences were found in the history of diabetes and alcohol consumption among the male subjects in the three study groups.

### 3.2 DVA and CAD prevalence association

**Figure 2:**
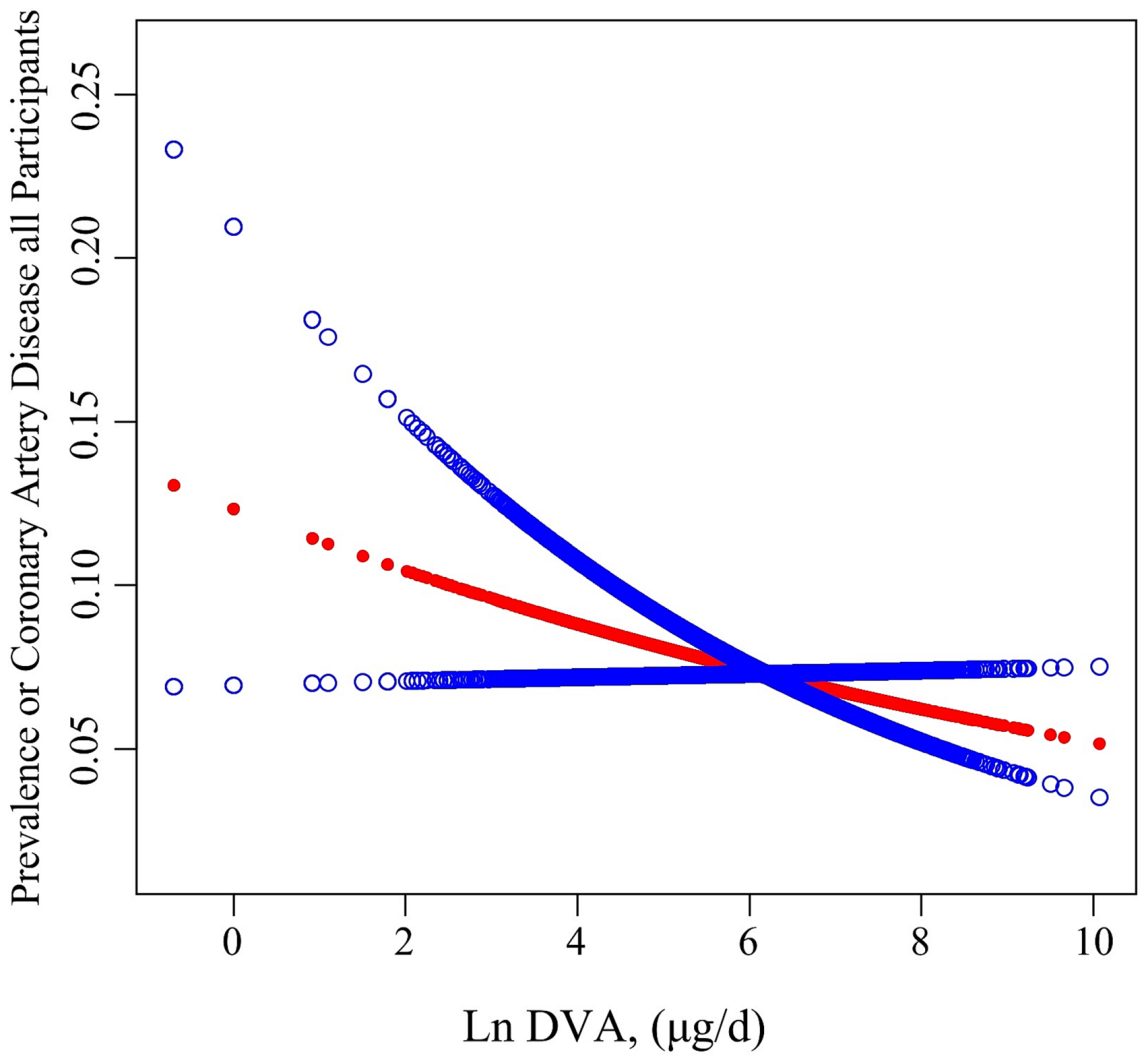
Association between DVA and the prevalence of CAD. The red line and blue line represent the estimated values and their corresponding 95% confidence intervals, respectively. Adjustment factors included age, sex, race, education levels, PIR, BMI, waist circumference, smoking and drinking history, hypertension, diabetes, TC, triglycerides, UA, SCR, eGFR, BUN, ALT, and energy.

**Figure 3:**
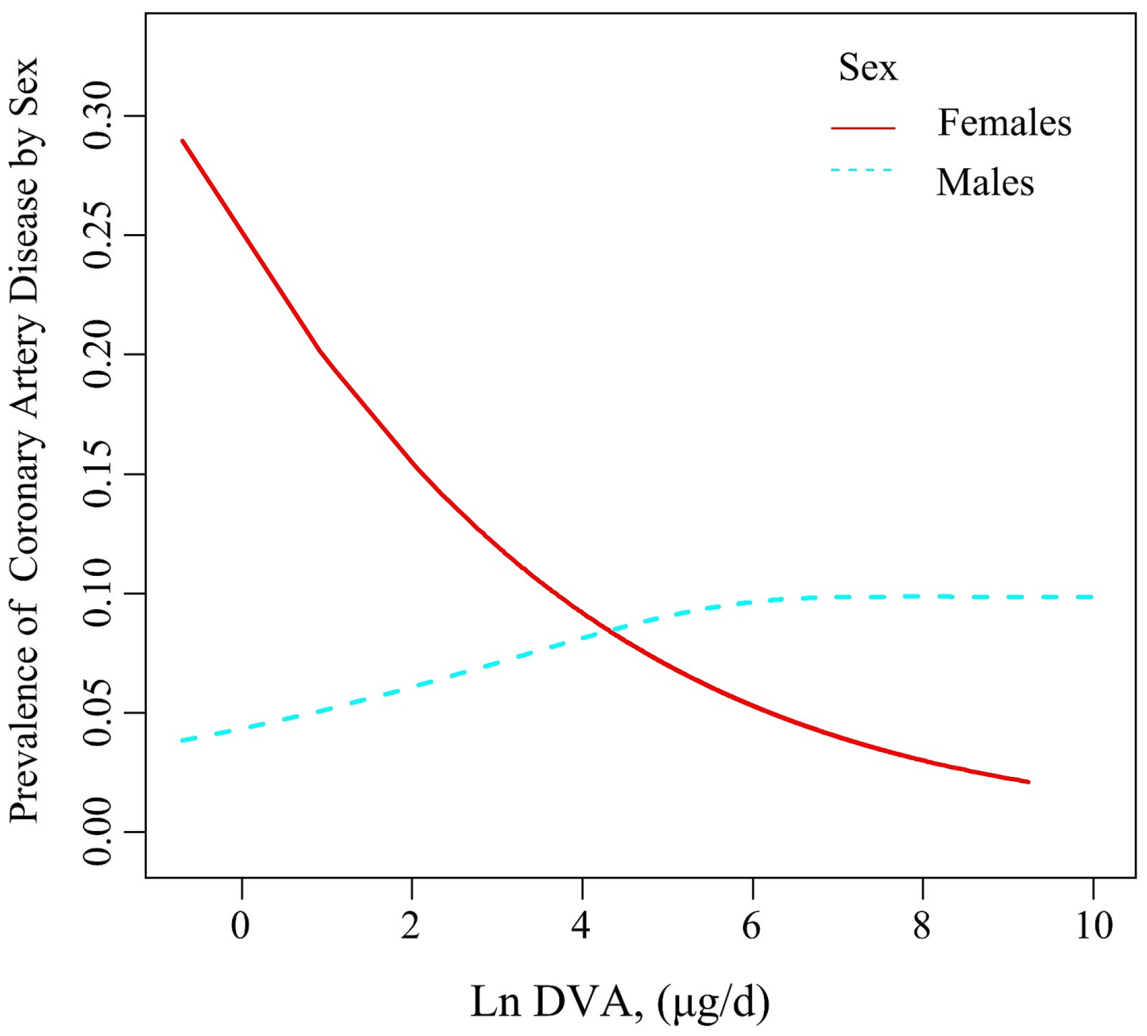
Association between DVA and the prevalence of CAD by sex. The solid line and dashed line represent the estimated values in males and females, respectively. The adjustment factors included age, sex, race, education levels, PIR, BMI, waist circumference, smoking and drinking history, hypertension, diabetes, TC, triglycerides, UA, SCR, eGFR, BUN, ALT, and energy.

Table 2 summarises the analysis of the association of DVA with CAD using multivariate regression, and Figure 2 characterizes the shape of the relationship after adjustment. Overall, adjusted DVA was linearly negatively correlated with CAD (per natural ln (DVA) increment: OR 0.91, 95% CI 0.83–0.99, *p* = 0.026). Furthermore, after converting the continuous DVA to tertiles of DVA, the ORs of adjusted tertile 2 (376.5–666.0, μg/d) and tertile 3 (666.5–23582.0 μg/d) were 0.91 (95% CI 0.79–1.05) and 0.83 (95% CI 0.71–0.97), respectively, compared to tertile 1 (0.0–376.0, μg/d). Generalized additive model and smoothed curve fitting showed that DVA and CAD prevalence were negatively correlated in female participants, but not in males (Figure 3). This is consistent with the results of multivariate regression analyses, in which the ORs for the association between DVA and CAD prevalence among female participants were remarkably stable (OR 0.79, 95% CI 0.72–0.87, OR 0.72, 95% CI 0.65–0.80, and OR 0.78, 95% CI 0.68–0.89 in Models 1, 2, and 3, respectively), and the trend tests were all statistically significant. However, among male participants, ORs were unstable and not statistically significant across models. Reanalysis of the interpolated data still yielded consistent results: among female participants, the fully adjusted pooled ORs for the relationship of DVA as continuous and tertile values with CAD were 0.79 (95% CI 0.68–0.91) and 0.79 (95% CI 0.62–1.00), respectively; however, among male participants, DVA was not significantly associated with CAD either as a continuous or a tertile value (Supplementary Tables S2, S3). Sex was able to modify the relationship between DVA and CAD prevalence (*p* for interaction <0.001).

**Table 2:** Relative odds of CAD according to DVA in different models among US adults.

Dietary vitamin A, μg/d	Event (%)	Coronary artery disease OR (95% CI), *p*-value		
		Model 1	Model 2	Model 3
All participants		*N* = 26,449	24,174	22,248
Continuous (ln DVA)	1,991 (7.53%)	0.95 (0.89, 1.01) 0.074	0.83 (0.78, 0.89) <0.001	0.91 (0.83, 0.99) 0.026
Tertile 1 (0.0–376.0)	669 (7.6%)	Reference	Reference	Reference
Tertile 2 (376.5–666)	684 (7.75%)	1.03 (0.92, 1.15) 0.623	0.85 (0.75, 0.96) 0.011	0.91 (0.79, 1.05) 0.217
Tertile 3 (666.5–23582.0)	638 (7.23%)	0.95 (0.85, 1.06) 0.356	0.70 (0.61, 0.80) <0.001	0.83 (0.71, 0.97) 0.017
*p* for trend		0.056	<0.001	0.017
Males		*N* = 12,748	11,660	10,802
Continuous (ln DVA)	1,255 (9.84%)	1.06 (0.98, 1.14) 0.162	0.92 (0.84, 1.00) 0.058	1.01 (0.90, 1.13) 0.897
Tertile 1 (0.0–396.0)	384 (9.04%)	Reference	Reference	Reference
Tertile 2 (396.5–710.5)	467 (10.99%)	1.24 (1.08, 1.43) 0.003	1.01 (0.86, 1.19) 0.863	1.03 (0.85, 1.22) 0.790
Tertile 3 (711.0–23582.0)	404 (9.51%)	1.04 (0.90, 1.21) 0.560	0.80 (0.67, 0.94) 0.009	0.92 (0.75, 1.13) 0.416
*p* for trend		0.666	0.001	0.224
Females		*N* = 13,701	12,514	11,446
Continuous (ln DVA)	736 (5.37%)	0.79 (0.72, 0.87) <0.001	0.72 (0.65, 0.80) <0.001	0.78 (0.68, 0.89) <0.001
Tertile 1 (0.0–359.0)	291 (6.38%)	Reference	Reference	Reference
Tertile 2 (359.5–630.0)	226 (4.95%)	0.79 (0.66, 0.94) 0.003	0.69 (0.57, 0.85) <0.001	0.82 (0.66, 1.01) 0.065
Tertile 3 (630.5–10475.5)	219 (4.79%)	0.74 (0.62, 0.88) 0.001	0.65 (0.54, 0.80) <0.001	0.79 (0.60, 1.00) 0.051
*p* for trend		<0.001	<0.001	0.025
*p* value for interaction^*^		<0.001	<0.001	<0.001

Values are ORs (95% CIs) unless otherwise indicated. CAD, coronary artery disease; DVA, dietary vitamin A; ln DVA value was log *e*-transformed. Model 1 was adjusted for none. Model 2 was adjusted for age, sex (only for the overall population), race, education levels, and PIR. Model 3 was adjusted for age, sex (only for the overall population), race, education levels, PIR, BMI, waist circumference, smoking and drinking history, hypertension, diabetes, TC, triglycerides, UA, SCR, eGFR, BUN, ALT, and energy. *p value for the interaction test: the two-way interaction of sex and Ln DVA (continuous) on CAD.

### 3.3 Subgroup analysis

**Figure 4:**
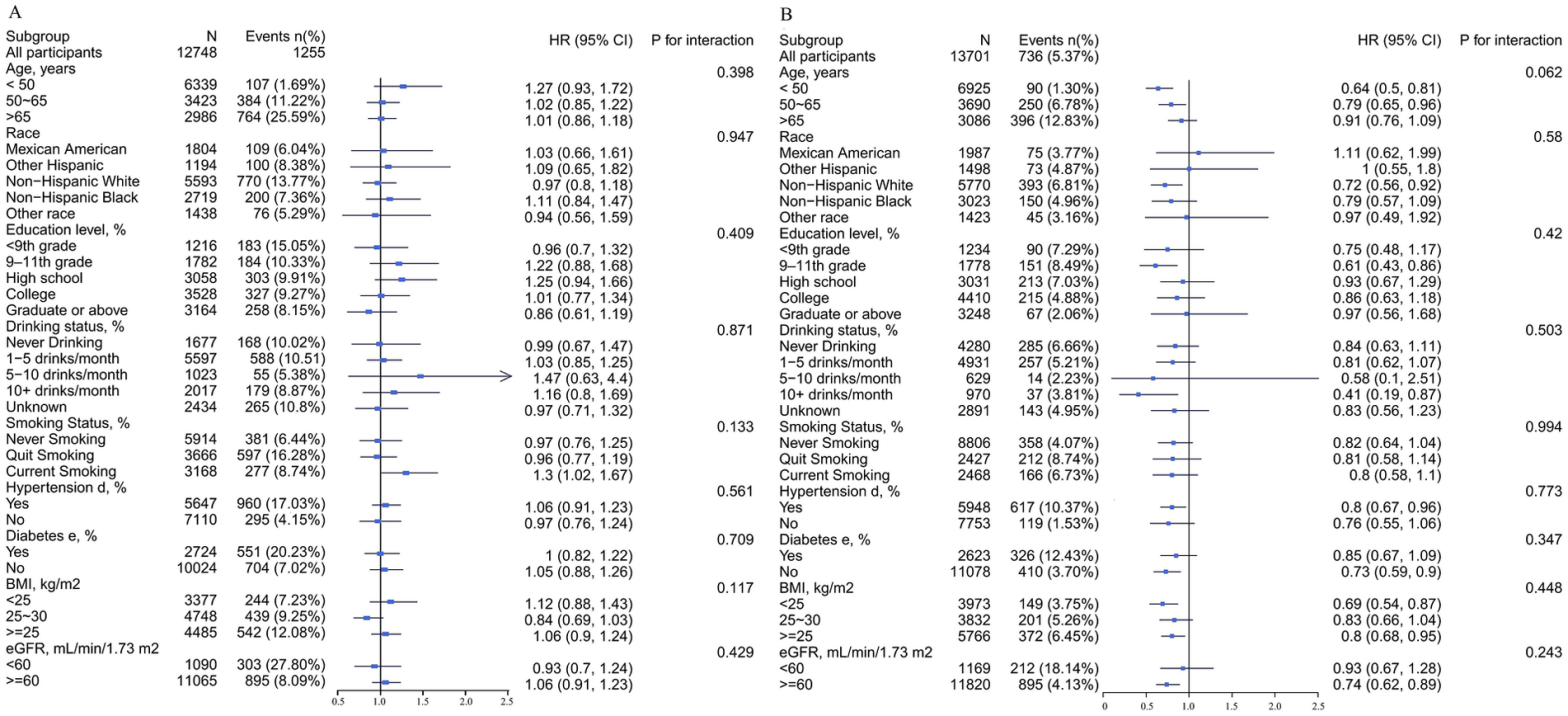
Stratified analyses by potential modifiers of the association between DVA and the prevalence of CAD by sex^*^. (A) Males and (B) females. ^*^Each subgroup analysis was adjusted for age, race, education levels, PIR, BMI, waist circumference, smoking and drinking history, hypertension, diabetes, TC, triglycerides, UA, SCR, eGFR, BUN, ALT, and energy. Except for the stratifying variable.

This study performed stratified analyses in male and female participants to assess the association of DVA with CAD prevalence in separate subgroups. In the whole population, we found that age significantly modified the relationship between DVA and CAD prevalence (*p* for interaction = 0.019), with a significant negative correlation between DVA and CAD in those <50 years of age and a neutral relationship in those >65 years of age. Similar findings were found for female participants but not for males. Other variables including race, education, smoking history, drinking history, hypertension, diabetes, and eGFR did not significantly alter the relationship between DVA and CAD prevalence in the entire population (Supplementary Figure S1), male participants (Figure 4A), or female participants (Figure 4B) (*p* for all interactions >0.05).

## 4 Discussion

Our large cross-sectional study, based on NHANES data between 2007 and 2018, found that DVA reduced the prevalence of CAD. Further analyses showed that appropriately increasing daily vitamin A intake only reduced the prevalence of CAD in the female population, while no such relationship was observed in the male population. In addition, this was the first study to assess sex differences in the relationship between DVA and CAD.

Previous studies have not evaluated the relation of DVA to the prevalence of CAD, and the majority of studies have observed an association of plasma vitamin A levels with CAD and its related conditions. An early case-control study involving 82 participants suggests that increased concentrations of vitamins A and E are independently related to a reduced risk of CAD in white South African men (18). Basnet et al. (19) also found vitamin A to be one of the important nutritional factors in CAD. Similarly, Matos et al. (20) found a correlation between vitamin A status and the severity of CAD. In addition, a large cohort study reported that adequate nutritional intake of vitamin A was associated with reduced all-cause or CVD mortality (21). However, an early multicenter, randomized, double-blind, placebo-controlled primary prevention trial involving 18,314 smokers, ex-smokers, and lime-exposed workers showed no benefit from additional supplementation with carotenoids and vitamin A and may have had a detrimental impact on the incidence of lung cancer and cardiovascular disease, as well as on all-cause mortality (9). Subsequently, to account for the increase in cardiovascular mortality, further analysis of the data on serum lipids from the study revealed that the mean cholesterol concentration in the group with additional carotenoid and vitamin A supplementation was 5.3 mg/dL (0.137 mmoL/L) above that of the placebo group (10). Taken together, the results of these studies are limited in addressing the relationship between vitamin A and CAD, and the relationship between DVA and CAD remains unclear.

Examining previous studies, this large-scale cross-sectional study we conducted is the first to assess the association of DVA with the prevalence of CAD among U.S. adults, filling a gap in the area and bringing some new findings. First, this study found that DVA was a protective factor for CAD: the prevalence of CAD gradually decreased with increasing DVA intake. Although the pathological mechanisms are unclear, they seem physiologically plausible. Atherosclerosis is a systemic inflammatory disease resulting in lipid deposition in the arterial wall due to endothelial dysfunction caused by a chronic inflammatory response (22). Oxidative stress is an important part of this process (23, 24). Vitamin A is a fat-soluble vitamin consisting of 3 active forms (retinol, retinaldehyde, and retinoic acid) with strong antioxidant properties (25). An intervention study that included 46 participants showed that vitamin A supplementation for 4 months could reduce inflammatory cytokine IL-17 production and gene expression of the major transcription factor controlling T-helper cells (Th17 cells) differentiation (26). In a similar study, Sezavar et al. (27) found that vitamin A supplementation inhibited T helper cells (Th1 cells) activity in both atherosclerotic and healthy participants. In addition, vitamin A is thought to be involved in ameliorating cardiovascular disease risk factors, including improving blood pressure, improving blood glucose lipid metabolism, reducing LDL oxidation, and inhibiting smooth muscle cell activity (28). Although the above findings appear encouraging, they are limited by sample size and population characteristics, so further long-term clinical studies are justified and necessary.

Second, the findings indicated that there was a significant interaction between sex and DVA, and sex could modify the association of DVA with CAD (*p* for interaction <0.001). Higher intake of vitamin A was associated with a lower prevalence of CAD in female participants, which was not the case in male participants. This discrepancy seems to be accounted for by sex hormone differences between men and women. Studies have shown that the incidence of cardiovascular disease in women is significantly lower than in men of similar age, and the risk of cardiovascular disease is higher in women with hyperandrogenemia (29–31). A study suggests that a diet rich in green lutein-carotenoid vegetables reduces the incidence of CAD in women, but not in men (32). Furthermore, retinoic acid is one of the most active forms of vitamin A. It plays an important role in cell growth, differentiation, and embryonic development processes (33). The production of retinoic acid begins with the metabolite of vitamin A, retinol, which is converted to retinaldehyde by the alcohol dehydrogenase family of enzymes, but this step is reversible (34, 35). The cytoplasmic aldehyde dehydrogenase 1 (ALDH1) family of enzymes catalyzes the oxidation of retinaldehyde to retinoic acid, completing this irreversible final step (35, 36). Evidence suggests that Aldh 1 activation levels, expression levels in different tissues, and effects on retinoic acid are regulated by sex hormones (37, 38). In animal models of atherosclerosis constructed through diet, males formed atherosclerotic plaques earlier and more broadly than females, and independent of lipid status (39, 40). The above results may be explained by the direct action of sex hormones within the vascular wall and the effect on cardiovascular risk factors. In addition, we note that age may be able to modify the relationship between DVA and CAD in female participants, which may also be due to the fact that younger women benefit from the protective effects of estrogen on the cardiovascular system, whereas after menopause, estrogen depletion leads to a higher risk of developing CAD (41). On the other hand, nutritional deficiencies continued to be a severe public health problem among women of childbearing age (15–49 years) (42). It has been reported in recent years that about one-third of women of childbearing age globally suffer from varying degrees of nutritional deficiencies (43). This deficiency affects maternal metabolism and tissue proliferation and hurts fetal growth and development (44). An epidemiological study showed that the prevalence of vitamin A deficiency in African women of childbearing age ranged from 4 to 22 percent (45). Therefore, an appropriate increase in vitamin A intake may be more beneficial to women. Furthermore, women consumed more vitamin A from vegetable sources and less from animals compared to men (46). Sex differences in the source of DVA may impact CAD development. Further, differences in lifestyle habits may also contribute to this outcome, with males more likely to be smokers and drinkers. Studies have shown that ethanol can compete with retinol for ethanol dehydrogenase, which catalyses the oxidation of retinol to retinaldehyde, which is then further oxidized to retinoic acid (47). In additional, studies have shown that the body converts beta-carotene to vitamin A at a maximum of 50 percent efficiently. Non-smokers, women and underweight people absorb the most beta-carotene, while smokers and alcohol drinkers absorb the least (48). However, more studies are necessary to validate our views and further explore their underlying mechanisms.

This study also has some limitations, which necessitates a well-designed large prospective study to validate the findings. Firstly, this study is a cross-sectional research design and the influence of unknown or unmeasured confounding factors (e.g., other nutritional factors, environmental exposures, etc.) on the results cannot be completely ruled out, nor can causal inferences be made. Secondly, we used dietary intake and outcome variable data obtained based on questionnaires, which might be prone to recall bias and measuring errors. Nevertheless, it is worth noting that questionnaires play a major role in national surveys of health and nutrition, and many high-quality studies have been performed on the basis of questionnaire data. In addition, caution is needed when extrapolating this result to other geographic areas due to geographic differences.

## 5 Conclusion

DVA was negatively associated with the prevalence of CAD, suggesting a protective effect of DDA against CAD. Further analysis revealed an interaction between DVA and sex in terms of CAD prevalence: in women, higher DVA was associated with a lower prevalence of CAD, whereas in men the two were not significantly correlated.

## Data Availability

The original contributions presented in the study are included in the article/Supplementary material, further inquiries can be directed to the corresponding authors.

## Supplementary material

SUPPLEMENTARY FIGURE S1 Stratified analyses by potential modifiers of the association between DVA and the prevalence of CAD for whole participants^*^. ^*^Each subgroup analysis was adjusted for sex, age, race, education levels, PIR, BMI, waist circumference, smoking and drinking history, hypertension, diabetes, TC, triglycerides, UA, SCR, eGFR, BUN, ALT, and energy. Except for the stratifying variable.
