# Supplementary figures and images for: Exploring the association between dietary vitamin A and coronary artery disease risk in men and women: findings from a US population study
Source: Front Nutr. 2024 Oct 15;11:1418159. doi: 10.3389/fnut.2024.1418159 (PMC11518770; doi:10.3389/fnut.2024.1418159)

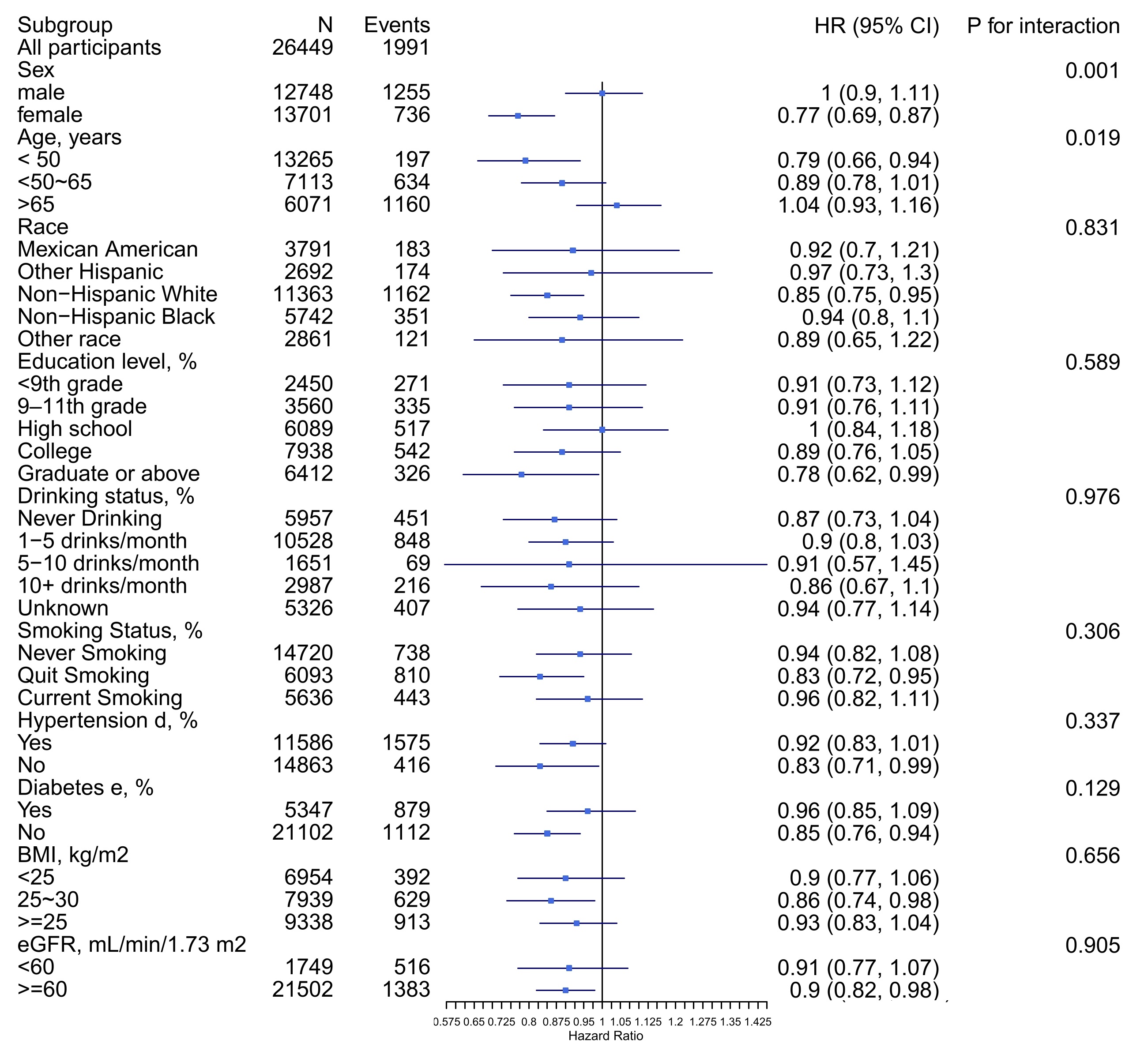

Supplement: SUPPLEMENTARY FIGURE S1 — Stratified analyses by potential modifiers of the association between DVA and the prevalence of CAD for whole participants*. *Each subgroup analysis was adjusted for sex, age, race, education levels, PIR, BMI, waist circumference, smoking and drinking history, hypertension, diabetes, TC, triglycerides, UA, SCR, eGFR, BUN, ALT, and energy. Except for the stratifying variable. [file Image_1.JPEG]
